# Abdominoperineal Resection for Rectal Cancer: Is the Pelvic Drain Externalization Site an Independent Risk Factor for Perineal Wound Healing?

**DOI:** 10.1155/2012/156935

**Published:** 2012-03-06

**Authors:** M. G. Pramateftakis, D. Raptis, D. Kanellos, E. Christoforidis, G. Tsoulfas, I. Kanellos, Ch. Lazaridis

**Affiliations:** ^1^4th Surgical Department, Aristotle University of Thessaloniki, G. Papanikolaou General Hospital, Exochi, 57010 Thessaloniki, Greece; ^2^Surgical Department, European Medical Center, Pilea, 55236 Thessaloniki, Greece

## Abstract

*Aim*. The aim of this paper is to investigate if the insertion of the pelvic drainage tube *via* the perineal wound could be considered as an independent risk factor for perineal healing disorders, after abdominoperineal resection for rectal malignancy. *Patients and Methods*. The last two decades, 75 patients underwent elective abdominoperineal resection for malignancy. In 42 patients (56%), the pelvic drain catheter was inserted through the perineal wound (PW group), while in the remaining 33 (44%) through a puncture skin wound of the perineum (SW group). Patients' data with respect to age (*P* = 0.136), stage (*P* > 0.05), sex (*P* = 0.188) and comorbidity (*P* = 0.128) were similar in both groups. 25 patients (*PW* versus *SW*: 8 versus 17, *P* = 0.0026) underwent neoadjuvant radio/chemotherapy. *Results*. The overall morbidity rate was 36%, but a significant increase was revealed in *PW* group (52.4% versus 9%, *P* = 0.0007). In 33.3% of the patients in the *PW* group, perineal healing was delayed, while in the *SW* group, no delay was noted. Perineal healing disorders were revealed as the main source of increased morbidity in this group. *Conclusion*. The insertion of the pelvic drain tube through the perineal wound should be considered as an independent risk factor predisposing to perineal healing disorders.

## 1. Introduction

The abdominoperineal resection (APR) was first described by Miles in 1908, but early clinical trials reported operative morbidity rates as high as 40% [1–4]. Nissan et al. [5] reported an overall morbidity rate of 50–60% in patients undergoing APR for carcinoma. After the rectum is excised, the sacral cavity forms a large wound area that cannot be efficiently reduced. That area is prone to retention and infection. Besides, it is well documented that postoperative complications of the perineal wound and their long-term residuals comprise the major morbidity factor, especially when combined with neoadjuvant radio/chemotherapy [6–8].

According to published data, some authors recommend the pelvic drain externalization through an abdominal stab incision, while others bring out the tubes directly through the perineum—either *via *a separate skin incision or *via *the perineal wound [9–11]. With regards to our technique, we believe that a perineal externalization site produces better results due to the gravity. To our knowledge, there are no studies up to date concerning the effects of the pelvic drain externalization site on the morbidity rates.

 The aim of this study is to investigate whether the insertion of the pelvic drainage tube *via* the perineal wound could be considered as an independent risk factor for perineal healing disorders, following APR for rectal malignancy.

## 2. Patients and Methods

Between 1991 and 2010, elective abdominoperineal resection for rectal carcinoma was performed in 75 patients (47 males and 28 females) with a mean age of 69 years (range, 22–82 years). The preoperative assessment for all patients included blood tests, chest X-ray, colonoscopy, and abdominal computed tomography. Since 1995, pelvic MRI was also routinely used for staging purposes. The mean distance of the tumors from the dentate line was 3.1 cm (max: 4 cm, min: 0.5 cm).

Preoperative bowel preparation with polyethylene glycol was routinely performed. Antibiotic prophylaxis consisted of intravenous 2nd generation cephalosporin and metronidazole, given at induction. During procedures lasting more than 2 hours, another dose was administered. No further postoperative antibiotics were used, unless a postoperative complication had arisen that needed treatment. All procedures were performed by one senior colorectal surgeon. Before the beginning of the procedure, randomization of the patient to either one of the two groups found place using a computer-generated ballot.

With regards to the technique used, we performed both abdominal and perineal approaches with the patient in modified lithotomy position. The abdomen was entered through a midline incision, extending from the pubis cephalad to just above the umbilicus. This approach allows adequate visualization of the abdomen, as well as the extension of the incision cephalad, should the splenic flexure need mobilization. A total mesorectal excision with high ligation of the inferior mesenteric vessels and preservation of the pelvic plexuses was performed.

Ninety-two percent of the interventions (69 patients; 38 of the *Perineal Wound* and 31 of the *Skin Wound* group) were performed with curative intent, whereas in 6 patients (8%), 4 of the PW and 2 of the SW group, the procedure was palliative. In one patient of the *Perineal Wound* group, the posterior vaginal wall was also resected *en bloc *with the rectum. In a further *Perineal Wound* group patient, two metastases of the right hepatic lobe were enucleated using radiofrequency ablation. With regards to the *Skin Wound* group, one patient underwent total hysterectomy and resection of the posterior vaginal wall, one underwent resection of the posterior bladder wall, and a third patient underwent resection of the posterior vaginal wall.

**Figure 1:**
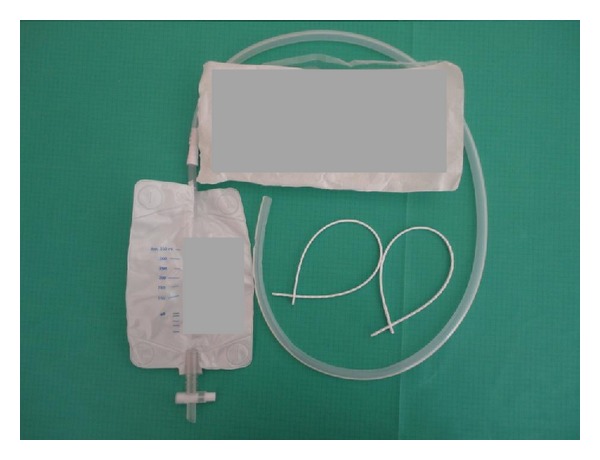
Type of a 30-Fr passive drainage system.

 A 30-Fr passive drainage was inserted and the peritoneal pelvic floor was reconstructed. This device, also known as gravity drainage system, consisted of a plain tube and a 350 mL volumetric bag (Figure 1). The perineal wound was primarily closed in a two-layer fashion. In 42 patients (56%), the pelvic drain was inserted through the perineal wound, whereas in the remaining 33 patients (44%), the drain was inserted *via* a puncture skin wound to the left lateral portion of the perineum. The puncture site used was due to surgeon's preference. Dermatological anomalies that would not allow the positioning of the puncture wound at this point were not observed in any patient. The pelvic drain was left *in situ *until either the daily fluid amount was less than 50 mL, or the drain had been *in situ* for 5–7 days and the patient was ready for discharge. In the latter case, the drain was removed irrespective of the daily output amount of the drain.

Twenty-five patients, 8 of the *Perineal Wound* and 17 of the *Skin Wound* group (*P* = 0.0026), underwent neoadjuvant chemoradiation. Surgery was performed six to eight weeks after preoperative radio/chemotherapy (Table 1). Patients' data with respect to age, sex, and comorbidity were similar in both groups (Table 2).

## 3. Statistical Analysis

Fisher's exact test was used for the comparisons between proportions. All the statistical analyses were performed using the SPSS v.15.0 statistical package (SPSS Inc, Chicago, IL, USA), enhanced with the modules exact tests.

## 4. Results

All patients were followed-up in our clinic on a weekly basis following their discharge for the first month and monthly thereafter. During follow-up, all patients had their baseline observations taken and a thorough examination of the perineal wound was performed by the operating surgeon and one assistant surgeon. Signs of localized infection, cellulitis, or delayed healing (such as redness, discoloration, swelling, warmth, etc.) were noted and recorded.

Most tumors in both groups were classified as BII according to Duke's classification and most of them were moderately differentiated. The detailed classification and differentiation of all tumors in the two groups is presented in Table 3.

Postoperative complications were observed in 22 patients of the *Perineal Wound* as well as in 5 patients of the *Skin Wound group. *With regards to surgery-specific complications, 20 were noted in patients of the* Perineal Wound* group, as well as 3 in patients of the *Skin Wound* group (PW versus SW: 47.6% versus 9%, *P* = 0.0002). The incidence of perineal wound healing disorders was significantly higher in the *Perineal Wound* group (PW versus SW: 33.3% versus 0, *P* < 0.001). In detail, 14 patients of the *Perineal Wound* showed a delay in perineal wound healing; in 11 of these patients, the perineal wound healing process was completed in 25–40 days (mean 31.2 days), while in three patients a permanent fistula was formed. In the *Skin Wound* group the mean time until complete perineal wound healing was 10 days and no case of healing disorder was noted. On the other hand, the number of patients who underwent neoadjuvant radio/chemotherapy was significantly higher in the *Skin Wound* group (SW versus PW: 51.5% versus 19%, *P* = 0.0026), and it is widely known that the incidence of wound healing abnormalities is reported to be higher in these patients. The rate of nonspecific, postoperative complications was exactly the same in both groups (Table 4).

The overall morbidity rate was 36%, but the statistical analysis revealed a significant increase in the *Perineal Wound* group (PW versus SW: 52.4% versus 9%, *P* = 0.0007). 5-year follow-up was completed for 49 patients, with a nonsignificant comparison between the study groups (SW versus PW: 26 versus 23, *P* = 0.1336). With regards to the survival rates, no significance was revealed after the pair-wise comparison (SW versus PW: 73.07% versus 73.9%, *P* = 0.253), while the overall rate was 73.4% (Table 5).

## 5. Discussion

The abdominoperineal resection of the rectum is one of the most demanding procedures in gastrointestinal surgery and has undergone only slight technical modifications since its first description [12–15].

In patients undergoing APR and especially for carcinoma, multiple specific complications may arise either in the short or long term. According to published data, the overall morbidity ranges from 50 to 60% after an APR [16]. Murrell at al. [17] reported that the most common immediate postoperative complication, with a frequency of 32%, is the formation of an intra-abdominal or pelvic abscess. In our study the incidence of this complication was extremely low, as only one case of abscess in the presacral space was noted, which was treated successfully with computed tomography-guided drainage and intravenous antibiotics. Other known complications include nerve injury, ureteric injury, complications from the colostomy site, as well as perineal wound complications [18, 19].

In the past, when blunt dissection was used with little appreciation to the fine pelvic anatomy, sexual dysfunction was seen in up to 75% of men and 40% of women, while bladder dysfunction was seen up to 80% of cases. Nowadays, following the introduction of TME, these rates—even though influenced by age, tumor location, and comorbidity—are reported to be 10–30% for sexual dysfunction and less than 5% for bladder dysfunction [20, 21]. Moreover, postoperative radiation tends to exacerbate male sexual dysfunction [22]. In our study, 2 cases (2.7%) of urinary but no case of sexual dysfunction was noted, as sharp dissection in the proper planes helped avoiding injury to the nerve plexuses.

The perineal wound poses a unique risk, predisposing to major postoperative complications. Despite improved surgical techniques, the rates of perineal wound dehiscence are reported to be higher than 10%, as it was also shown in our data. Furthermore, it is observed in 30–40% of patients who undergo neoadjuvant radiation [23–26]. The anatomy of the pelvic floor and the inherent potential risk of infection secondary to rectal surgery are associated with a high rate of perineal healing abnormalities following an APR. Besides, perioperative chemoprophylaxis fails to provide sufficient protection, because vessel ligature and electrocoagulation result in reduced perfusion and consequent disorders in microcirculation of the sacral cavity [27].

A confounding issue is the different opinions as to what risk factors impair the perineal wound healing. According to Christian et al. [28], higher rates of major wound complications were associated with increased body mass index, diabetes, and stage, while preoperative radiation and primary closure were not associated with increased complications. On the other hand, Luna-Pérez et al. [29] demonstrated that the main cause of morbidity was perineal wound infection, influenced by postoperative radio +/− chemotherapy administration and patient age over 55 years.

In our patient group, the overall morbidity rate was 36%, while perineal healing disorder was noted to be the most common postoperative complication (59.3% of all case complications). Primary healing of the perineal wound, meaning no formation of seroma or hematoma and no signs of inflammation, was seen in 78.7% of patients. In the *Perineal Wound* group, the morbidity was significantly higher compared to the *Skin Wound* group (52.4%; 22/42 of patients, *P* = 0.0007). Perineal wound healing abnormalities were the main source of increased morbidity in this group (72.8%; 16/22 of complicated cases, *P* = 0.001). There were 14 cases (33.3%) of delayed perineal healing, as well as 2 cases (4.8%) of perineal wound infection/dehiscence, which were treated conservatively.

Apart from the pelvic drain externalization site, patients in both groups showed no statistically significant differences with regards to population data, comorbidities, disease stage, and intraoperative conditions. As mentioned before, these parameters have been reported to affect perineal wound healing in many publications [30, 31]. Moreover, it is shown that even though the number of patients who underwent neo-adjuvant radiation was significantly higher in the *Skin Wound* group, the rate of perineal wound healing abnormalities was significantly lower in these patients compared to those of the *Perineal Wound* group. This fact correlates with recently published studies suggesting the lack of any relation between pelvic irradiation and perineal healing abnormalities [26]. According to these findings, it is clearly demonstrated that the insertion of the pelvic drain tube through the perineal wound constitutes an independent risk factor affecting perineal wound healing, which results in increased postoperative morbidity rates in patients undergoing APR for rectal cancer.

## 6. Conclusion

The insertion of the pelvic drain tube through the perineal wound should be considered as an independent risk factor following an APR, predisposing to perineal healing disorders.

## Figures and Tables

**Table 1:** Patients treated by APR for a low rectal cancer (*n* = 75).

Group	PW (*n* = 42)	SW (*n* = 33)	*P* value	Significance level
Age*	67.2 (22–81)	71.3 (41–80)	0.157	NS
Sex ♂/♀	26/16	21/12	0.1878	NS
Tumor loc.**		3.1 (0.5–4)		
Indication				
Curative	38 (90%)	31 (94%)	*0.2935*	NS
Palliative	4 (10%)	2 (6%)	*0.2935*	NS
Neo-adjuvant RT/CT	*8*	*17*	*0.0026*	Sig.
Adjuvant RT/CT	21	8	*0.0147*	Sig.

*yrs: value I median (range).

**cm from the dentate line: value is mean (range).

(NS: non-significant; *P* value >0.05; Sig.: significant, *P* value <0.05).

**Table 2:** Risk factors associated with increased morbidity after APR; comparison of the study groups.

Group	PW (*n* = 42)	SW (*n* = 33)	*P* value	Significance level
Age (>55 yrs)	29	26	0.136	NS
Comorbidity	30	27	0.128	NS
Diabetes	11	7	0.191	NS
Cardiopulmonary dis.	4	5	0.212	NS
Vascular dis.	8	9	0.153	NS
Obesity (B.M.I. >30 kg/m^2^)	12	9	0.203	NS
Neo-adjuvant RT/CT	*8*	*17*	*0.0026*	Sig.

NS: non-significant, *P* value >0.05; Sig.: significant, *P* value <0.05.

**Table 3:** Staging and differentiation.

Group	PW (*n* = 42)	SW (*n* = 33)	*P* value	Significance level
Staging (Duke's)				
In situ	2	—	*0.310*	NS
A	3	3	*0.311*	NS
BI	6	5	*0.254*	NS
BII	14	13	*0.165*	NS
CI	3	3	*0.311*	NS
CII	11	7	*0.191*	NS
D	3	2	*0.351*	NS
Differentiation				NS
Well	10	4	*0.107*	NS
Moderate	28	23	*0.189*	NS
Poor	4	6	*0.149*	NS

NS: non-significant, *P* value >0.05.

**Table 4:** Complications, morbidity, and mortality.

Group	PW (*n* = 42)	SW (*n* = 33)	*P* value	Significance level
Complications	22	5	*0.0007*	Sig.
Surgical (specific)	20	3	*0.0002*	Sig.
Abdominal wound dehiscence	2	2		
Pelvic abscess*	1	—		
Ostomy necrosis**	—	1		
Evisceration/reoperation	1	—		
Perineal healing disorders	**16**	—	***<0.001***	Sig.
Delay in perineal healing	14	—		
Perineal wound dehiscence	2	—		
Medical (nonspecific)	2	2	*0.374*	NS
Pneumonia	1	—		
Urinary dysfunction	1	1		
Atrial fibrillation	—	1		
Morbidity	**52.4%**	**15.2%**	***0.0007***	Sig.
Mortality		0		
Overall morbidity		**36%**		

*In the presacral space, treated with CT-guided drainage.

**treated with primary relocation.

(NS: non-significant, *P* value >0.05; Sig.: significant, *P* value <0.05).

**Table 5:** Local recurrence and survival.

Patients (*n*)	SW	PW	Overall		*P* value*	Significance level
Compl. 5-year follow-up	26	23	49		0.1336	NS
Deaths	7	6	13		0.2831	NS

*Cause of death*				*Time after APR (months)*		

LR**	1	1	2	12, 24		
LR** + hepatic metastases	—	1	1	6		
Hepatic metastases	3	2	5	12, 12, 14, 18,38		
Brain metastases	1	—	1	24		
Lung + hepatic metastases	1	1	2	18, 24		
Hepatic + brain metastases	1	—	1	24		
Stroke	—	1	1	36		

Survival (%)	73.07	73.9	***73.4***		0.253	NS
Local Recurrence (%)	3.8	8.7	***6.1***		0.357	NS

*SW versus PW.

**LR: local recurrence.

NS: non-significant, *P* value >0.05; Sig.: significant, *P* value <0.05.
